# A Highly Sensitive and Room Temperature CNTs/SnO_2_/CuO Sensor for H_2_S Gas Sensing Applications

**DOI:** 10.1186/s11671-020-3265-7

**Published:** 2020-02-14

**Authors:** Yang Zhao, Jijun Zhang, Yan Wang, Zexiang Chen

**Affiliations:** School of Optoelectronic Science And Engineering, University of Electronic Science and Technology of China, North Jianshe Road 4, Chengdu, 610054 China

**Keywords:** H_2_S detection, Room temperature sensor, SnO_2_ nanocomposite, Gas sensor

## Abstract

Gas sensors based on tin dioxide-carbon nanotube composite films were fabricated by a simple inexpensive sol-gel spin-coating method using PEG400 as a solvent. Nanostructured copper was coated on CNTs/SnO_2_ film, and then copper was transformed into copper oxide at 250 °C. Resistivity of the final composite films is highly sensitive to the presence of H_2_S, which became easily attached or detached at room temperature. The response and recovery time of the sensor are 4 min and 10 min, and the value of sensitivity is 4.41, respectively. Meanwhile, the CNTs/SnO_2_/CuO sensor also has low detection limit, high selectivity toward H_2_S, and stable performance with different concentrations of H_2_S.

## Introduction

With the development of industrialization, emission pollution is becoming increasingly serious, so different types of gas sensors have been widely studied [1–7]. SnO_2_ as a n-type and environment-friendly semiconductor has been studied by many different researchers [8–11]. It may be considered to be an excellent gas-sensitive material widely used for developing gas sensors because of its capacity to absorb molecules in the gas phase. The mechanism of gas detection is the change of material conductivity caused by the reversible gas-solid interaction on the surface of tin dioxide [12]. There are some methods that have been adopted to improve the performance of SnO_2_ gas sensor, including doping with metallic oxide (e.g., TiO_2_, La_2_O_3_) [13, 14], catalytic active additives (e.g., Pt, Pd, and Au) [9, 15–18], and addition of graphene and carbon nanotubes [8, 19, 20]. It has applications in environmental problems and industrial gas-monitoring issues, such as SO_2_ [21], CO [20, 22], NO_2_ [23], and H_2_S [24, 25], which represent a great concern for environmental safety.

Hydrogen sulfide is a colorless, toxic gas. There are many sources of hydrogen sulfide, usually as a product of the natural decomposition process of certain chemical reactions and protein and some impurities which exist in various kinds of production processes, such as the mining and non-ferrous metal smelting, sulfur-oil exploration, rubber and sugar industry, excavation of low-temperature coking coal, and treatment of swamps, canals, and sewers. Hydrogen sulfide is a harmful gas for human health [26–31]. Even low concentrations of hydrogen sulfide also can damage the human sense of smell. High concentrations of hydrogen sulfide can paralyze olfactory nerves [30, 32]. As a means of detecting the gas with the nose is deadly, the detection of hydrogen sulfide is necessary.

Studies demonstrate that carbon nanotubes (CNTs) are good candidates as potential “dopants” of SnO_2_ [19, 33]. CNTs have a large surface area and it is easy for molecular adsorption [34]. And CNTs can also influence the material’s charge transfer electrostatic environment, thus improving the performance of SnO_2_ sensors.

Gas sensors based on SnO_2_ have been shown to detect nitrogen dioxide, carbon monoxide, liquefied petroleum gas [35], volatile organic compound gases, and other gases and vapors. However, in order to obtain reasonable responses, the operating temperatures of these sensors conventionally need to be above 200 °C. There are barely reasonable responses at room temperature. Frank et al. [19] develop a gas sensor for H_2_S at room temperature with tin dioxide-carbon nanotube composite films. Although the response and recovery time of the gas sensor is much short, the sensitivity is low.

In this paper, a novel gas sensor based on carbon nanotube-tin dioxide (CNTs/SnO_2_) composite films with nano-copper oxide have been successfully synthesized. The sensor can detect H_2_S with low concentration with the response time down to dozens of seconds. The most important is the sensitivity is much higher than other gas sensors at room temperatures.

## Experimental Section

### Materials and Methods

**Fig. 1:**
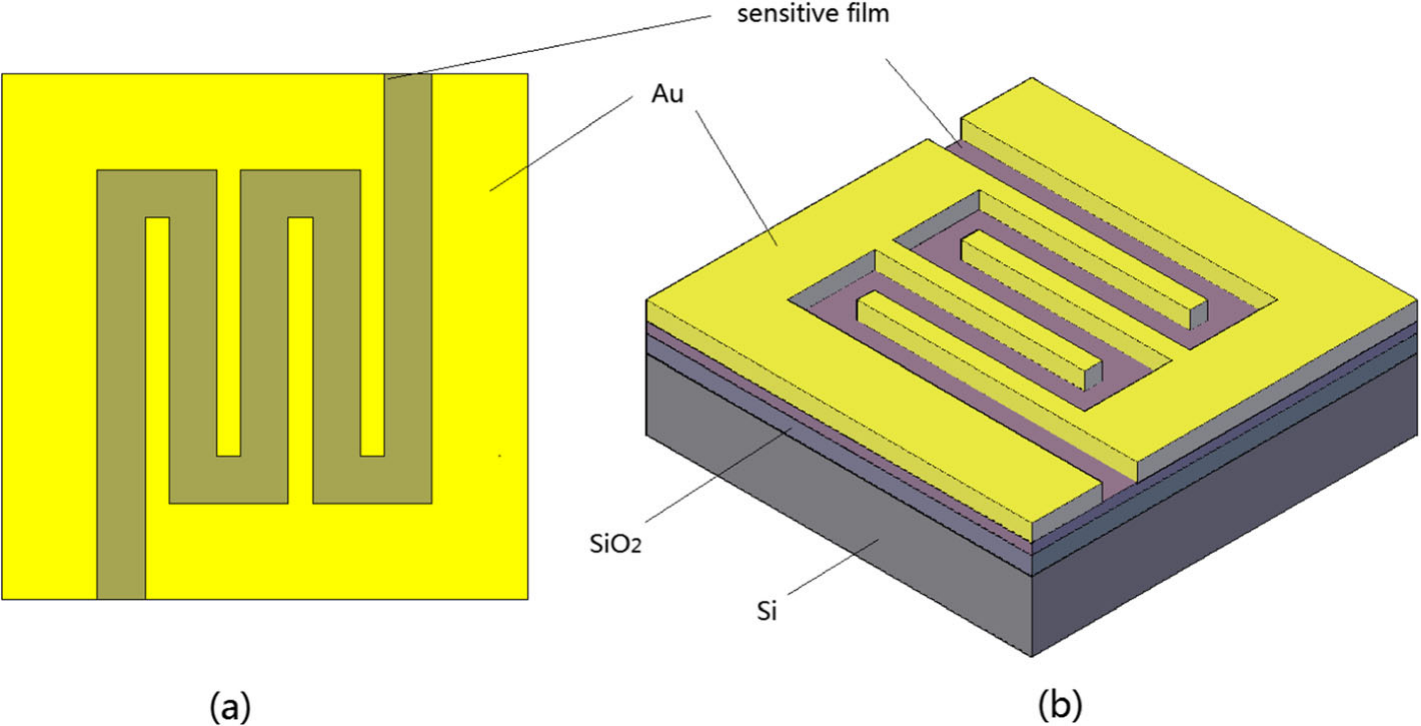
Schematics of **a** top view of gas sensor and **b** stereogram of gas sensor

The CNT was purchased from Chengdu Organic Chemicals Co. Ltd., Chinese Academy of Sciences. We treated carbon nanotubes for acidification with volume ratio for 3:1 of concentrated sulfuric acid and concentrated nitric acid. Firstly, the SnCl4, as a raw material for the SnO_2_ by sol-gel, was dissolved in the EG at the temperature of 80 °C with magnetic stirring. The CNT was added to the above solution and magnetically stirred at 80 °C for 3 h. Later, the temperature was up to 120 °C for hydrolysis reaction about 3 h. After that, the PEG-400 was added to the solution with magnetic stirring in order to make it easy for acquiring surface films. The spin-coating method has been used to form the composite surface films in silica substrates. We used a tube furnace for heating treatment at 450 °C for an hour of the spin-coating films that are to form the CNTs/SnO_2_ composite films. Thereafter, nanostructured copper about 6 nm was coated to the composite films with vacuum evaporation method with a current of 23 Å and the evaporation rate of 2 Å/s. Then copper was transformed into copper oxide at 250 °C for about 2 h. At last, we used vacuum evaporation to form the gold interdigital electrodes. The sample of the sensor is presented in Fig. 1. We also prepared for SnO_2_ and SnO_2_/CuO nanocomposite in the same way for the comparison.

### Gas Sensing Procedure

**Fig. 2:**
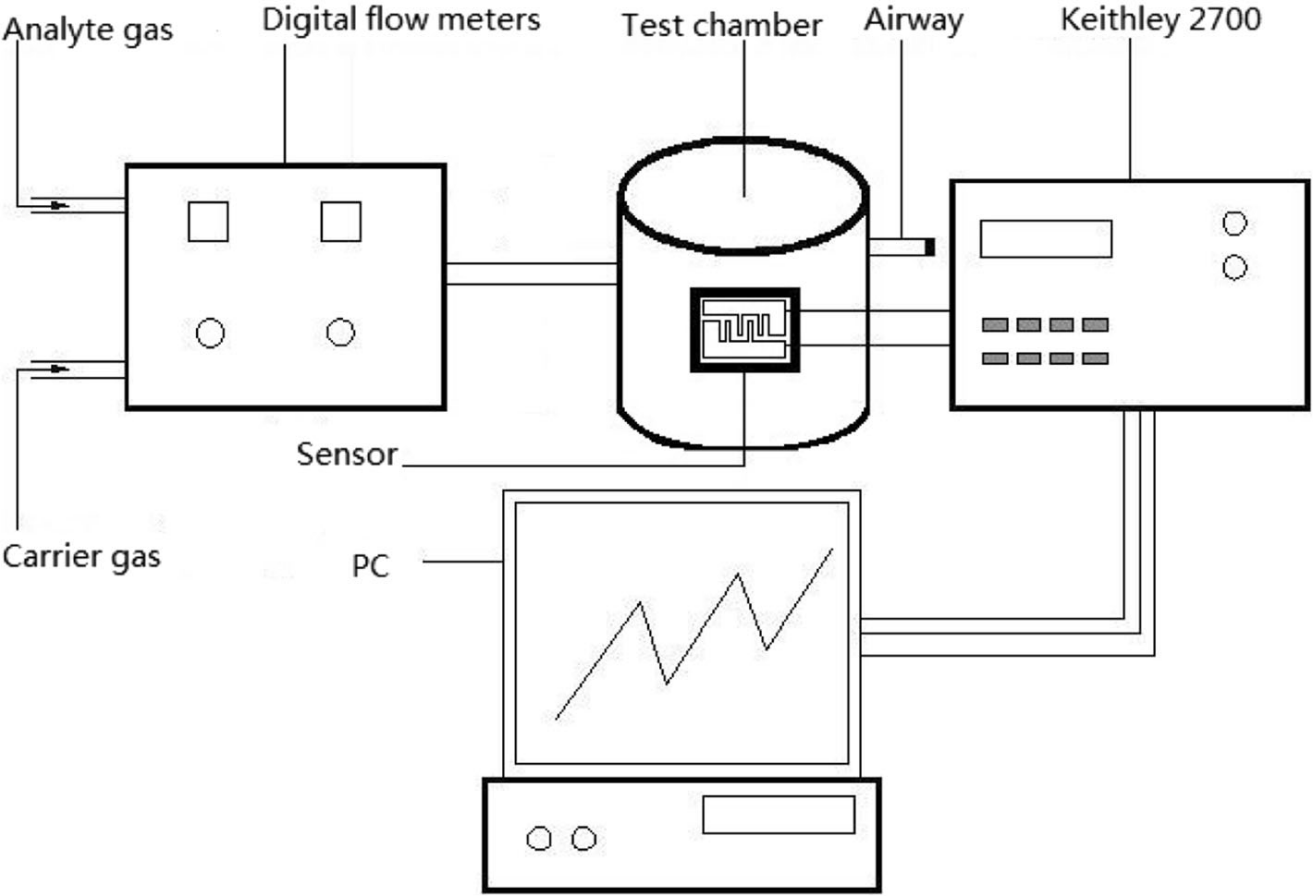
The setup employed for gas detection

Figure 2 shows the system for gas detection and nitrogen was used as the carrier gas. The original concentration of H_2_S is 1000 ppm. The required test concentration was obtained by the mixture of carrier gas and analyte gas so that the required ppm level can be attained. The concentration of analyte gas was precisely controlled by digital flow meters which controlled the flow rate of carrier gas and analyte gas. It maintains at a flow rate of 400 sccm when the gas passed through the test chamber. And the flow rates of carrier gas and analyte were changed in order to get the needed concentration by the digital flow meters. The standard of gas testing was carried out at room temperature, atmospheric pressure, and nitrogen gas atmosphere with negligible relative humidity.

The sensor was kept in the test chamber which was made by Teflon. The test chamber consists of four extraction electrodes, gas access, and airway. It can be placed over four sensor samples in the test chamber, so we can test four sensors at the same time. The Keithley 2700 was applied to detect the change of the resistance of the sensors. Communicating with computer through software, the real-time data will be displayed directly on the computer.

The resistance of the sensors can be acquired from Keithley 2700 in real-time. The curve of the change of the resistance can be obtained and displayed in the computer screen. The following are the formulas of gas response and sensitivity.

1 $$ \mathrm{Sensitivity}=\frac{R_a-{R}_c}{\Delta  C}=\frac{\Delta  R}{\Delta  C} $$

2 $$ \mathrm{Response}=\frac{R_a-{R}_c}{R_c}=\frac{\Delta R}{R_c} $$

where *R*_*c*_ represents the resistance of the device in a carrier gas of pure N_2_, *R*_*a*_ is the resistance of the mixture of carrier gas and analyte gas, and ∆*C* is the change of the analyte gas concentration, respectively.

## Results and Discussion

**Fig. 3:**
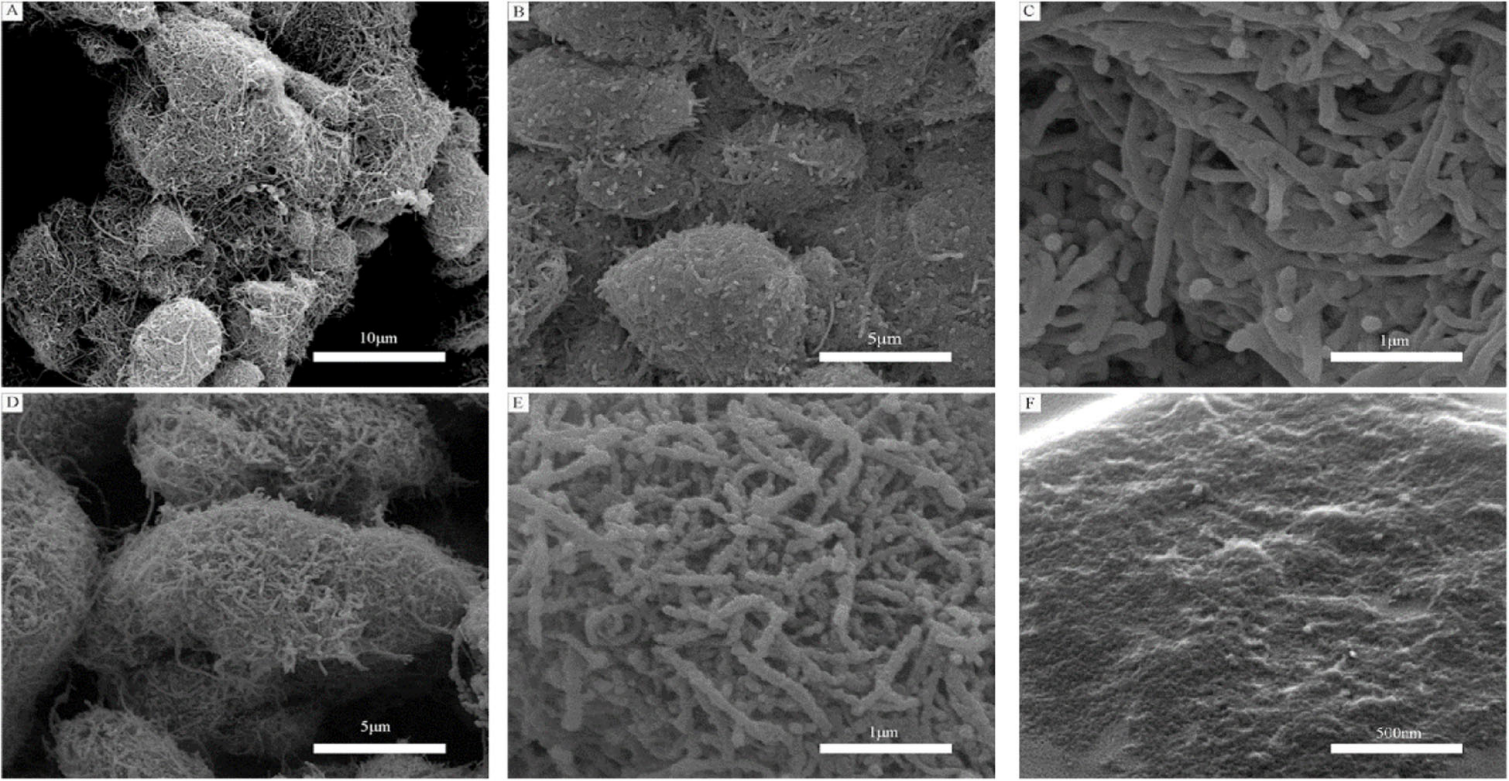
FESEM images of **a** pristine carbon nanotubes; **b**, **c** carbon nanotubes with oxidation treatment; **d**, **e** CNTs/SnO_2_ nanocomposite; and **f** CNTs/SnO_2_/CuO nanocomposite film

FESEM was carried out to obtain the morphological characteristics of samples as shown in Fig. 3. Figure 3 a shows the pristine carbon nanotubes; in the figure, they got together into a group and the structure is very intensive which gases can hardly get into the inside carbon nanotubes. And there are many impurities on the surface of carbon nanotubes. From Fig. 3 b and c, the impurities have disappeared and the carbon nanotubes became looser after oxidation treatment. The powder of the first step material CNTs/SnO_2_ was collected and the FESEM image of it is shown in Fig. 3 d and e. The carbon nanotubes which can be found in the figure have been a bit thicker and coarser compared to the carbon nanotubes in Fig. 3 c. It is a tin oxide coating to the carbon nanotubes. As shown in Fig. 3 f, the prepared composite film has been investigated. Porous and well-loose structure has been seen on the surface. It is likely to form a core-shell structure which is the carbon nanotubes as nuclear, the tin oxide, and copper oxide as a shell. And the carbon nanotubes in these areas perhaps play a role of transmission charge.

**Fig. 4:**
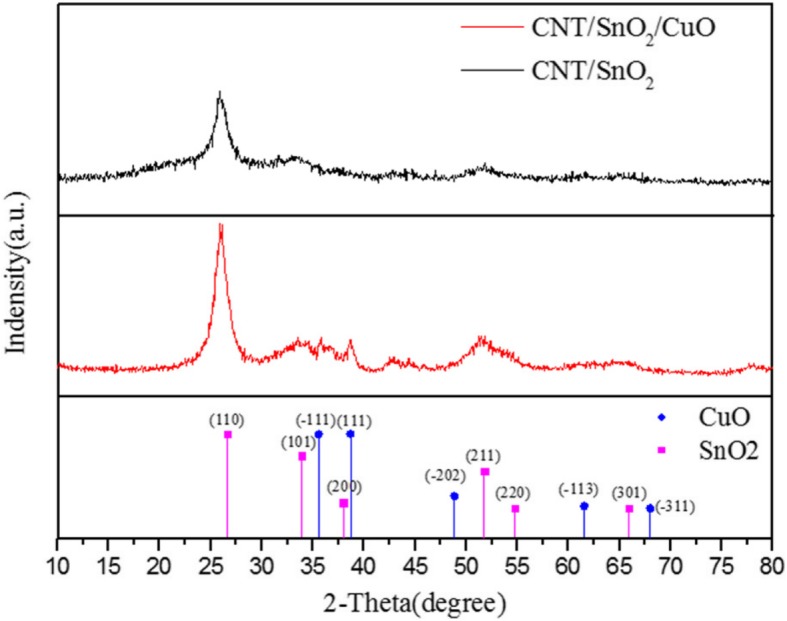
XRD patterns of the CNTs/SnO_2_ and CNTs/SnO_2_/CuO nanocomposite

The prepared samples were examined by XRD characterization and the XRD curves are shown in Fig. 4. We can clearly see an obvious peak at 2θ of 26° which is typical XRD peak for CNTs. Besides, the diffraction peaks at 26.6°, 33.8°, 51.8°, 54.7°, and 65.9° are indexed to the SnO_2_ (JCPDS card no. 41-1445). And because the content of CuO is too low, the peaks of CuO are not obvious. But we can still find the weak peaks at 35.5°, 38.6°, 48.8°, 61.5°, and 66.3° indexing to CuO (JCPDS card no. 89-2529).

**Fig. 5:**
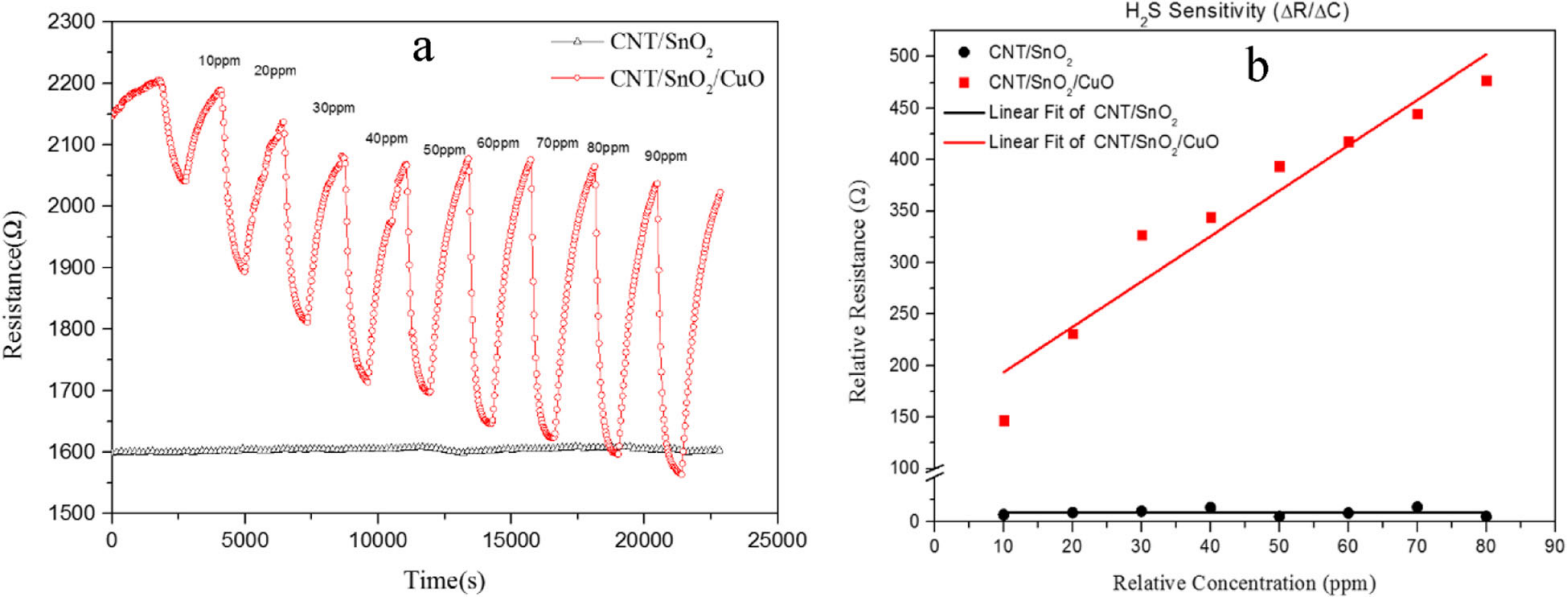
**a** The response of the CNTs/SnO_2_ and CNTs/SnO_2_/CuO nanocomposite to H_2_S. **b** The sensitivity of the CNTs/SnO_2_ and CNTs/SnO_2_/CuO to H_2_S

The response of the CNTs/SnO_2_ nanocomposite and the CNTs/SnO_2_/CuO nanocomposite to hydrogen sulfide is shown in Fig. 5 a with concentrations of 10, 20, 40, 60, and 80 ppm. When sensor materials were exposed to different concentrations of H_2_S at room temperature, they show the behavior of the resistance signal (response) as a function of the time [19]. Notice that the CNTs/SnO_2_ nanocomposite basically shows no response. Although there are some small differences about the baseline resistance of the concentration from 20 to 40 ppm, the CNTs/SnO_2_/CuO nanocomposite mainly keeps the good reversibility. It can be seen from the diagram that when the H_2_S gas is released into the test chamber (gas on), the response time is 4 min. Analogously, while the H_2_S gas is removed from the test chamber (gas off), the resistance increases with a recovery time of 10 min. The response time and recovery time are defined as the time taken for the sensor output to reach 90% of the highest response or 90% of minimum, respectively. In Fig. 5a, we can also obtain that as the concentration of the H_2_S gas increased, the variation of the resistance reduced. It may be the reason that the sensor is reaching saturation concentration as the gas concentration increased. Figure 5 b shows the H2S sensitivity values of the CNTs/SnO2 and CNTs/SnO2/CuO obtained from Eq. (1). From the plots, the relationship between relative resistance (ΔR) and relative concentration (ΔC) is an approximate linear. The sensitivity value of CNTs/SnO2/CuO is 4.41, while CNTs/SnO2 is 5.95 × 10−4. Compared with CNTs/SnO2, the sensitivity of the CNTs/SnO2/CuO nanocomposite material shows greatly improved at room temperature.

**Fig. 6:**
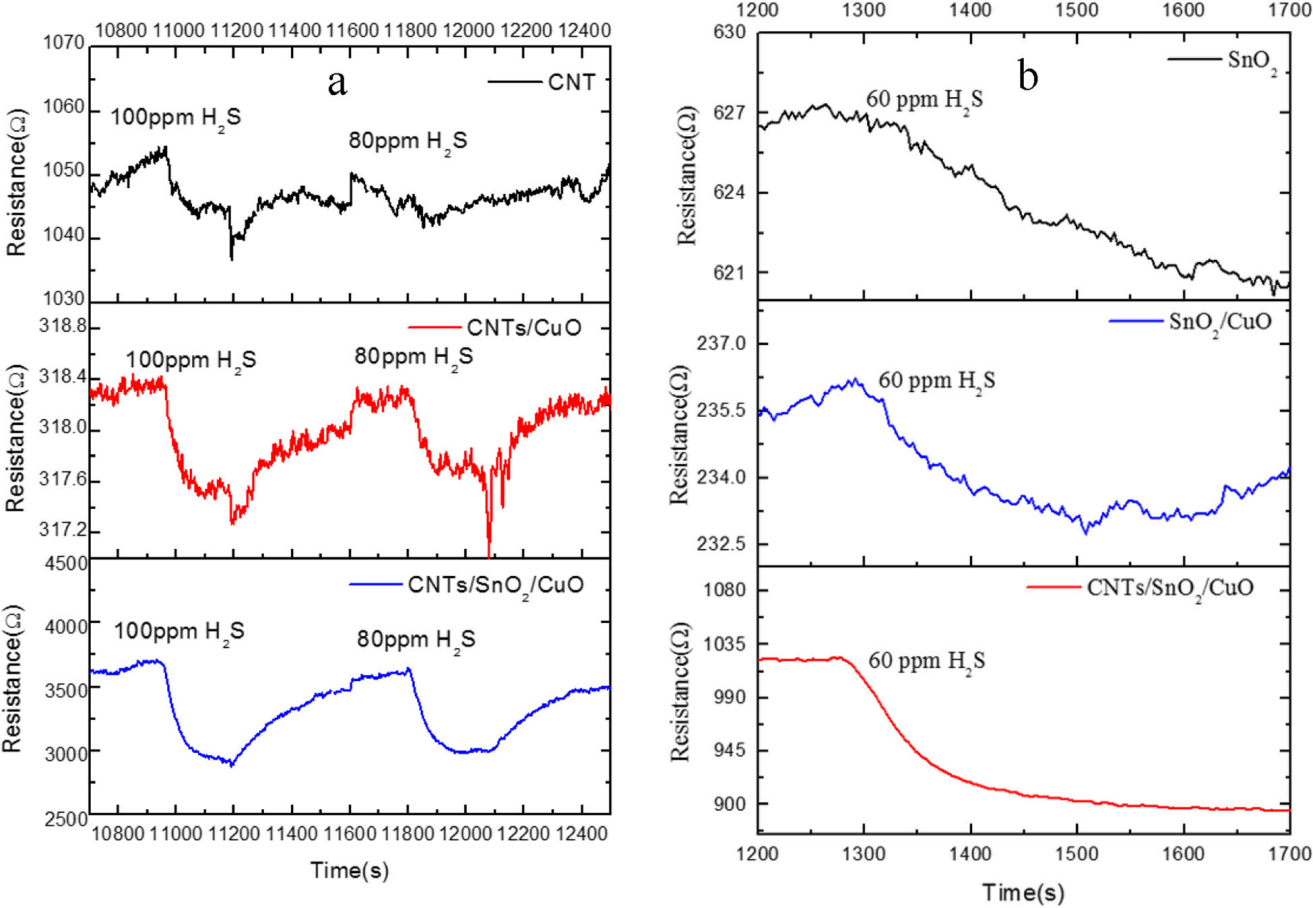
**a**, **b** Comparison of performance of nanocomposite in detecting H_2_S

Besides, the comparison of the performance of SnO_2_, CNTs/CuO, SnO_2_/CuO, and CNTs/SnO_2_/CuO nanocomposite in detecting H_2_S is shown in Fig. 6. It shows that the CNTs/SnO_2_/CuO-based sensor has the smoother response curves which mean less disturbances. Meanwhile, CNTs/SnO_2_/CuO-based sensor shows more sensitive in detecting H2S.

**Fig. 7:**
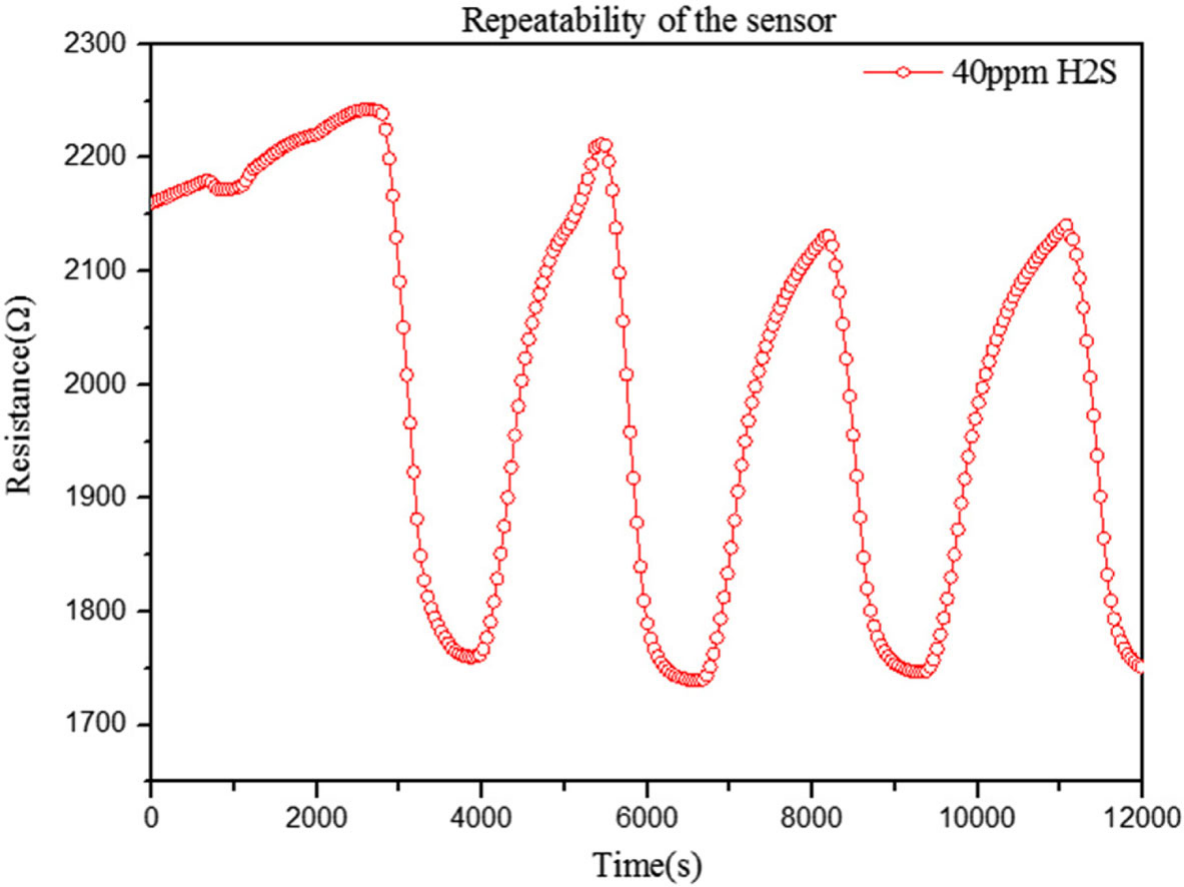
Repeatability of the sensor of CNTs/SnO_2_/CuO in the concentration of 40 ppm H_2_S

To investigate the repeatability of the sensor, we test the response and recovery characteristics at 40 ppm H_2_S and room temperature, as shown in Fig. 7. The curve indicates that the sensor of the CNTs/SnO_2_/CuO has good repeatability and stability in the concentration of 40 ppm H_2_S. The first reversible cycle of the response has some disturbances in the recovery region. It may be the reason that the baseline of the resistance of the sensor was not very smooth. As the time goes by, the resistance baseline became much smooth so that the later reversible cycle of the response and recovery curve became much better. The response and recovery time of the sensor may be a little longer than some of the sensors which may be related to some factors including the thickness of the sensing layer, the gas diffusion, and the amount of gas adsorption on the sensing material at different operating temperatures [36–38]. The sensor of the CNTs/SnO_2_/CuO may be the operating temperatures of room temperatures. At room temperature, the inorganic chemical reaction may be a little slow which makes the results. In another reason, it may be the high sensitivity which needs time to absorb gas and release gas.

**Fig. 8:**
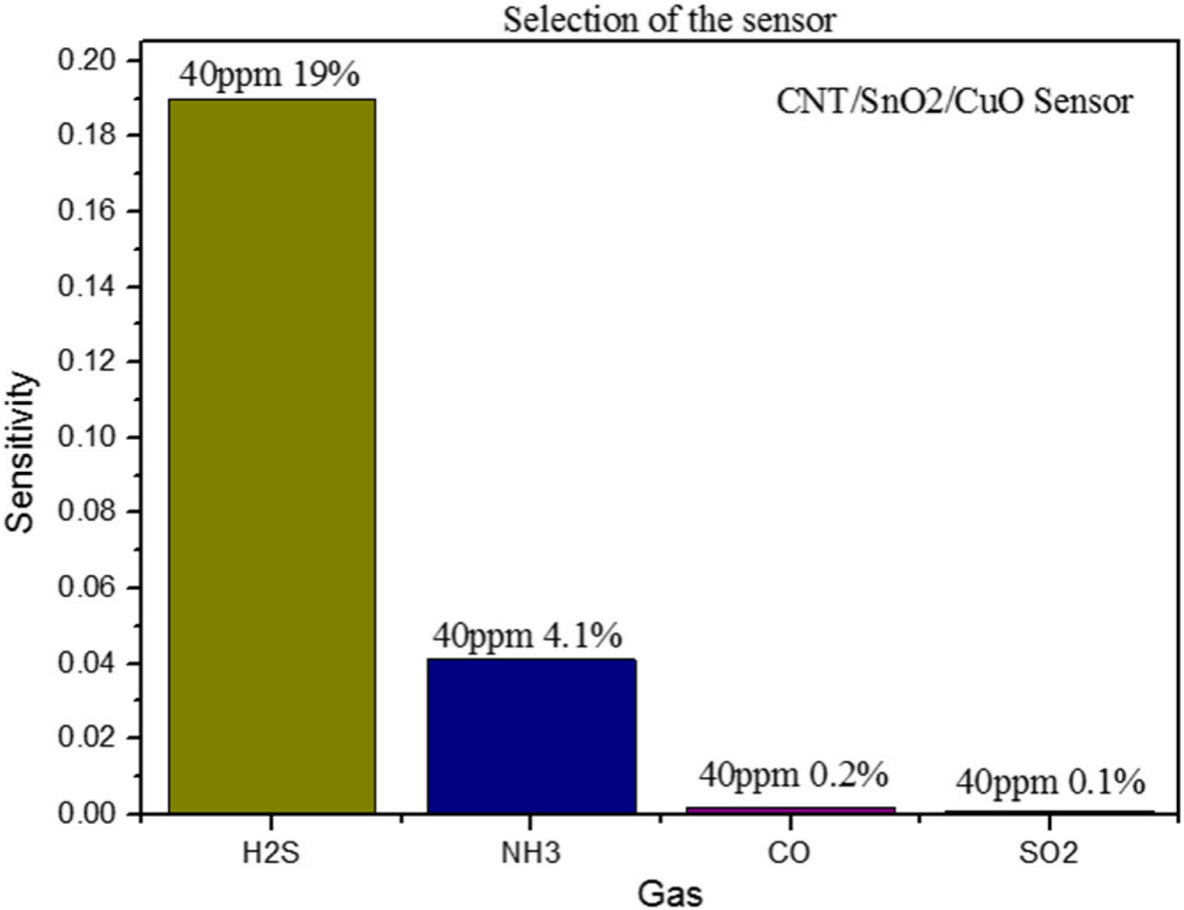
Selectivity of the sensor in 40 ppm for H_2_S, NH_4_, CO, and SO_2_

Figure 8 shows the bar diagram that illustrates gas selectivity of the CNTs/SnO_2_/CuO sensor at 40 ppm towards four gases. Obviously, it is seen that the sensitivity of the sensor to H_2_S is 19% which is the maximum response of the four gases. In addition, the sensitivity of the sensor to NH_3_ is 4.1% which is the second maximum response. And the sensitivity of the other two gases is much lower than the former which is nearly no response. It is revealed that the sensor has more excellent selectivity towards H_2_S than CO, SO_2_, and NH_3_. And it all comes down to different gases that have different energies when reacting with sensor materials. The reaction of H_2_S molecules with the CNTs/SnO_2_/CuO material could be faster and more responsive. The CNTs/SnO_2_/CuO sensor shows the most sensitive to H_2_S compared with other gases.

**Fig. 9:**
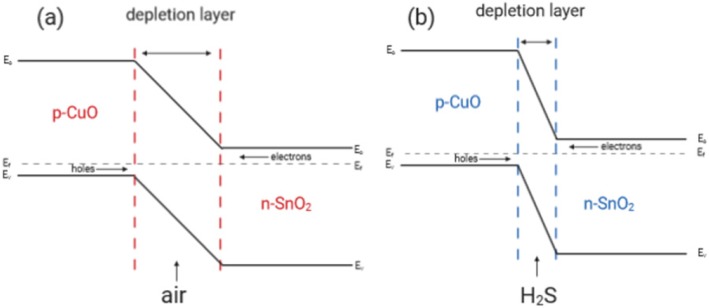
**a**, **b** Sensing mechanisms of SnO_2_/CuO heterojunction for detecting H_2_S gas

It was found that CNTs/SnO_2_/CuO-based sensor shows a large decrease in resistance of thin films on exposure to H_2_S gas. There are two main reasons that can explain the sensitive and selective detection mechanism of CNTs/SnO_2_/CuO nanocomposites. First of all, the core-shell structure of CNTs/SnO_2_ nanocomposites provides a larger surface area to adsorb and diffuse the gas molecules. Then the key to improving performance of gas sensing is the formation of p–n heterojunction between SnO_2_ and CuO. The p-CuO/n-SnO_2_ interface will form a charge carrier depletion layer which causes high resistance of sensing materials in air, as shown in Fig. 9a. When exposed to H_2_S gas, CuO was transformed to CuS, which breaks the p–n heterojunction. Thus, as shown in Fig. 9 b, the depletion layer becomes thinner and leads to low resistance of sensing materials.

## Conclusions

In summary, the CNTs/SnO_2_/CuO nanocomposite has been synthesized by a simple inexpensive way. And the sensor that use the CNTs/SnO_2_/CuO nanocomposite as the active material has been developed and tested at standard conditions at room temperature. The sensor has fast response (4 min) and recovery (10 min) at room temperature. And the CNTs/SnO_2_/CuO gas sensor could detect H_2_S concentration as low as 10 ppm. Meanwhile, the CNTs/SnO_2_/CuO gas sensor shows better performance than that of the CNTs/SnO_2_ sensor. Besides, the sensor has good repeatability and stability in the concentration of 40 ppm H_2_S and has more excellent selectivity towards H_2_S than other gases. Therefore, the CNTs/SnO_2_/CuO gas sensor is useful in many situations at room temperature, such as industrial safety.

## Data Availability

All data is fully available without limitations.

## Abbreviations

CNTs: Carbon nanotubes
EG: Ethylene glycol
FESEM: Field emission scanning electron microscopy
PEG: Polyethylene glycol
XRD: X-ray diffractometer
